# Adult-onset acquired port-wine stain in the absence of trauma: A case report

**DOI:** 10.1016/j.jdcr.2026.05.002

**Published:** 2026-05-11

**Authors:** Asem Mohammed Almesfer, Zubair Abdullah Wani, Abdulaziz Saud Alobaid, Mishari Tariq Alrubaiaan, Wafa Thyab Alanazi

**Affiliations:** a Department of Dermatology, King Fahad Medical City, Riyadh, Saudi Arabia; b Department of Dermatology, College of Medicine, King Saud bin Abdulaziz University for Health Sciences, Riyadh, Saudi Arabia; c Department of Dermatology, Northern Border Health Cluster, Ministry of Health, Arar, Northern Borders Province, Saudi Arabia

**Keywords:** acquired port-wine stain, adult-onset port-wine stain, fegeler syndrome, nontraumatic port-wine stain, vascular malformation

## Introduction

Port-wine stains (PWS), currently termed port-wine capillary malformations (PWCMs) according to the International Society for the Study of Vascular Anomalies classification, are congenital capillary malformations that present at birth as a pink-to-red patch that darkens over time.^1^^,^^2^ In contrast, acquired PWS or adult-onset PWS occur later in life. Trauma has been the most commonly reported trigger and is often described under the term Fegeler syndrome.^3^ Acquired PWS is a rare and infrequently reported condition, with a limited number of cases reported in the literature.^4^ Despite the delayed onset, it is morphologically and histologically indistinguishable from congenital PWS. Acquired PWS cases arising in the absence of an identifiable traumatic trigger are even more uncommon and may pose a diagnostic challenge.^4^ In this report, we discuss a case of acquired PWS in a 25-year-old male with a history of testicular mixed germ-cell tumor, contrasting with trauma-dominant profiles in the existing literature, highlighting the need to consider other nontraumatic causes.

## Case presentation

A 25-year-old male presented with an asymptomatic, progressively enlarging erythematous patch that had appeared 8 months earlier. The lesion originally appeared on the left posterior scalp and extended to encompass the left shoulder and upper back. The patient denied any history of similar cutaneous findings during childhood, confirming the acquired nature of the lesion. The patch progressively darkened and expanded over time without associated pain, pruritus, bleeding, or friability.

Further history revealed no preceding mechanical or thermal trauma, infection, or topical exposure. The patient was previously diagnosed with a right testicular mixed germ-cell tumor, consisting of 80% embryonal carcinoma and 20% yolk sac tumor, for which he underwent radical orchiectomy and refused to undergo adjuvant chemotherapy or radiotherapy 10 months prior to the appearance of the skin lesion. The patient was not on any regular medications and did not have a family history of neurocutaneous syndromes or vascular malformations.

**Fig 1:**
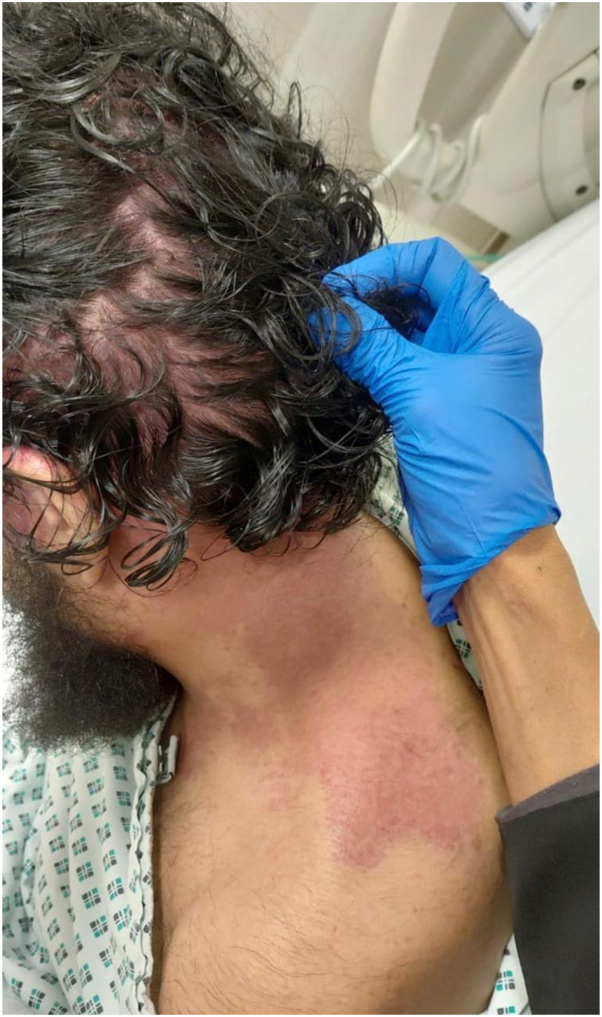
A well-demarcated erythematous to violaceous patch involving the left posterior scalp with extension to the posterior neck and upper back. The lesion is flat, nonblanchable, and shows no nodularity, or hypertrophy.

The clinical examination revealed a well-defined erythematous to violaceous patch on the left posterior scalp and extending to the left shoulder and upper back. The lesion was non-blanchable, smooth, with no evidence of induration, nodularity, or hypertrophy (Fig 1).

**Fig 2:**
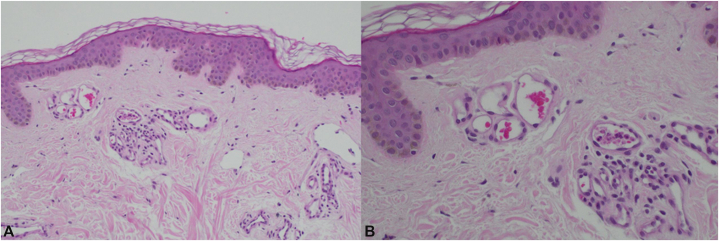
**A** and **B,** The biopsy shows dilated, thin-walled capillaries and venules predominantly in the superficial dermis, favoring port-wine stains.

Histopathologic examination of the skin punch biopsy demonstrated numerous dilated, thin-walled capillary and venular channels confined to the superficial dermis, consistent with PWS (Fig 2).

Given the absence of trauma and the patient's oncologic history, the findings support a diagnosis of a rare adult-onset acquired PWS. The patient was counseled on the benign nature of the condition and offered pulsed dye laser therapy for cosmetic improvement; however, he preferred to postpone treatment. The patient was followed for 3 months, during which the lesion remained clinically stable without progression or development of new symptoms.

## Discussion

Acquired PWS primarily has a clinical diagnosis that must be confirmed by ruling out conditions that mimic vascular stains. The primary differential diagnosis for adult-onset vascular stains is early inflammatory morphea. Before hardening and atrophy development, early morphea can present as an erythematous patch. Several cases have been reported of morphea being initially misdiagnosed as acquired PWS before the appearance of fibrosis months to years later.^5^ In our case, histopathologic examination revealed telangiectasia without collagen thickening, sclerosis, or inflammatory infiltrate, thereby ruling out morphea. Other histopathologic findings, including simple capillary dilation, helped rule out differentials such as tufted angioma and Kaposi sarcoma.^6^ Moreover, the lack of palpable induration on examination further supported the diagnosis of acquired PWS.^5^ Although the lesion remained stable during short-term follow-up, longer-term follow-up is warranted to definitively exclude the potential evolution into morphea.

The most recognized etiology of acquired PWS is trauma, initially defined by Fegeler in 1949.^3^^,^^7^ Skin injury damages the perivascular sympathetic nerve fibers responsible for vasoconstriction, resulting in persistent capillary ectasia that resembles a PWS.^7^ However, in this report, the patient denied any history of a preceding trauma. Given the absence of preceding trauma, this case represents a nontraumatic acquired PWS. The patient’s history of mixed germ-cell tumor further raised consideration of a potential paraneoplastic association. Even though rare, acquired vascular malformations have been associated with internal malignancies and hormonal fluctuations.^4^ Two similar cases have been described in the literature. Bansal et al documented a case of acquired PWS in an adult male with no identifiable etiology, despite the patient having a history of an underlying malignancy.^4^ Another report, Kulac et al described a case of acquired PWS associated with an acoustic neuroma.^8^ Though speculative, it is biologically plausible to state that systemic angiogenic factors or hormonal shifts associated with malignancies or their treatments could trigger vascular anomalies in susceptible individuals.^4^^,^^8^

Despite the patient’s oncological history, he did not receive systemic chemotherapy or radiotherapy. Although chemotherapy-induced vascular toxicity has been reported, including with agents such as bleomycin and cisplatin, this mechanism is unlikely in our case, as the patient was not exposed to those agents.^9^^,^^10^ Therefore, treatment-related vascular injury cannot account for the development of the lesion.

In either case of PWS, congenital or acquired, loss of neural regulation of blood flow is involved. In congenital cases, this is often driven by a somatic mutation in the Guanine nucleotide-binding protein G(q) subunit alpha (*GNAQ*) gene.^2^ On the other hand, it is suggested that postnatal factors disrupt the sympathetic nerve plexus surrounding the capillaries for the acquired form.^11^ However, somatic genetic analysis was not performed in this patient, and therefore the underlying molecular mechanism remains uncertain, representing a limitation of this report. Since this patient's lesion appeared after the tumor, the diagnosis suggests a potential disruption of autonomic regulation. Such a disruption could be secondary to the systemic effects of his illness, although a direct link remains unproven. Physicians must maintain a heightened level of suspicion for underlying etiologies in individuals exhibiting new vascular symptoms. Additionally, medical practitioners should carefully examine patients to eliminate mimics, such as early morphea, through biopsy and follow-up.

## Conflicts of interest

None disclosed.
